# Valorization of Purple Phototrophic Bacteria Biomass Resulting from Photo Fermentation Aimed at Biohydrogen Production

**DOI:** 10.3390/molecules29071679

**Published:** 2024-04-08

**Authors:** Grazia Policastro, Alessandra Cesaro, Massimiliano Fabbricino

**Affiliations:** 1 Department of Engineering, Telematic University Pegaso, 80143 Naples, Italy; grazia.policastro@unipegaso.it; 2 Department of Civil, Architectural and Environmental Engineering, University of Naples Federico II, 80125 Naples, Italy; massimiliano.fabbricino@unina.it

**Keywords:** anaerobic processes, hydrogen, microbial proteins, PHB, resource recovery

## Abstract

This study evaluated the feasibility of contextually producing hydrogen, microbial proteins, and polyhydroxybutyrate (PHB) using a mixed culture of purple phototrophic bacteria biomass under photo fermentative conditions. To this end, three consecutive batch tests were conducted to analyze the biomass growth curve and to explore the potential for optimizing the production process. Experimental findings indicated that inoculating reactors with microorganisms from the exponential growth phase reduced the duration of the process. Furthermore, the most effective approach for simultaneous hydrogen production and the valorization of microbial biomass was found when conducting the process during the exponential growth phase of the biomass. At this stage, achieved after 3 days of fermentation, the productivities of hydrogen, PHB, and microbial proteins were measured at 63.63 L/m^3^ d, 0.049 kg/m^3^ d, and 0.045 kg/m^3^ d, respectively. The biomass composition comprised a total intracellular compound percentage of 56%, with 27% representing PHB and 29% representing proteins. Under these conditions, the estimated daily revenue was maximized, amounting to 0.6 $/m^3^ d.

## 1. Introduction

Sustainable development is driving a rapid transition in the wastewater and organic waste treatment sectors, shifting from a “removal and disposal” strategy to the recovery and reuse of energy and materials. In this context, Purple Phototrophic Bacteria (PPB) are gaining increasing attention due to their metabolic adaptability, allowing for the valorization of waste/by-products for the recovery of energy and natural biobased products [1]. PPB are photosynthetic microorganisms capable of converting light into chemical energy. They thrive under anaerobic conditions, utilizing both organic and inorganic carbon sources and light as an energy source. Moreover, they are able to grow both anaerobically and aerobically (as chemotrophs) [2]. During photoautotrophic growth, PPB can fix inorganic carbon from dissolved carbon dioxide (CO_2_) and bicarbonate (HCO_3_^−^), bypassing the use of oxygen as an electron acceptor to avoid inhibiting photosynthetic complex formation. Alternatively, in the photoorganoheterotrophic metabolism, PPB preferentially utilize organic compounds as carbon and electron sources [1]. Unlike chemoorganoheterotrophic organisms, which require significant organic catabolism to produce ATP, photoorganoheterotrophs derive energy directly from light, leading to high biomass yields over organic substrates. Under this mode, PPB use organics as electron donors and biomass as electron acceptors in balanced conditions [3]. In addition to these metabolic pathways, PPB can activate photo fermentation (PF) metabolism, particularly under nitrogen scarcity and carbon excess, promoting hydrogen production [4]. This pathway involves substrate degradation primarily as a dissipative mechanism with redirected electrons toward hydrogen formation instead of biomass, via enzymes like nitrogenase and hydrogenase [2].

The metabolic versatility of PPB makes them suitable to produce various high-value products such as hydrogen and biomass-linked products, like polyhydroxyalkanoates (PHAs) and microbial proteins. These products have promising applications, including as renewable energy sources, biodegradable plastics, and alternative protein sources, contributing to sustainable resource management [1].

The majority of literature studies on PPB have utilized pure cultures focusing on specific metabolic pathways (photo fermentation aimed at hydrogen production or photoorganoheterotrophy for biomass production). For instance, *Rhodobacter* and *Rhodopseudomonas* species, *Rhodospirillum rubrum* and *Rhodovulum sulfidophilum*, have been extensively studied for their ability to produce high rates (from 0.4 to 3.2 mM/h) of molecular hydrogen [5]. *Rhodopseudomonas palustris* and *Rhodobacter capsulatus* have been reported to produce PHAs from 30 to 90% of their CDW under photoorganoheterotrophic growth [6]. Concerning the production of proteins, to date, most of the highest protein yields (74–90%) among PPB have been associated with *Rhodopseudomonas palustris* [7].

Although most studies on PPB have utilized pure cultures focusing on specific metabolic pathways and, therefore, specific products, recent research suggests that mixed and non-aseptic cultures can simultaneously produce hydrogen and PHAs, thanks to diverse microbial species within consortia [8,9]. This approach not only enhances product diversity but also facilitates scalability, as mixed cultures are more robust and adaptable to varying conditions. However, the simultaneous production of proteins alongside hydrogen and PHAs has not been explored in the existing literature, representing a significant research gap. Proteins, being one of the most valuable products from biomass, present promising opportunities for further investigation and exploitation.

This study evaluated the feasibility of producing hydrogen, proteins, and polyhydroxybutyrate (PHB) under photo fermentative conditions. After assessing biomass growth through sequential batch experiments, the final photo fermentation test utilized exponentially growing biomass enriched from previous batches to optimize the simultaneous production of hydrogen, PHB, and microbial proteins in terms of biomass quality, output productivity, and economic viability.

## 2. Results and Discussion

**Figure 1:**
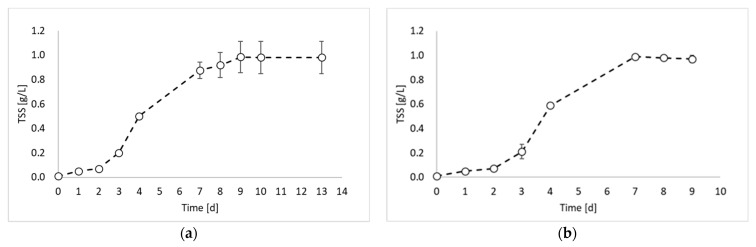

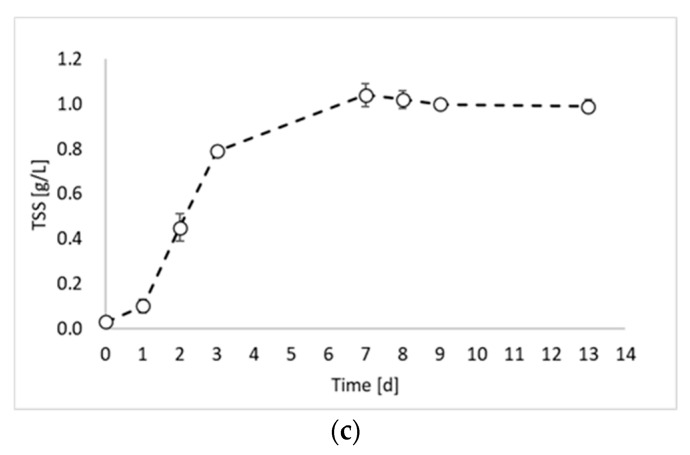
Total suspended solids (TSSs) concentration over time obtained during: (**a**) biomass growth, (**b**) inoculum production, and (**c**) hydrogen production tests.

In this study, the valorization of PPB biomass was studied under a photo fermentation condition. Figure 1 reports the biomass growth curves obtained from the biomass growth, inoculum production, and hydrogen production experiments.

Figure 1 shows that the maximum TSS value was similar for all experiments and ranged between 0.98 and 0.99 g_TSS_/L. This result was due to the experimental conditions, which were identical in all experimental tests (except the inoculum). During the first experiment (Figure 1a), a lag phase of about 2 days occurred, followed by an increase in the TSS values until day 9. After day 9, it is possible to observe the stationary phase. The maximum biomass growth rate was observed between day 2 and day 3 with a value of 0.0124 g_TSS_/L h. The second experiment (Figure 1b) showed very similar results, with a lag phase of two days and a maximum growth rate of 0.0145 g_TSS_/L h between day 3 and day 4. Conversely, a significant improvement in the biomass growth was obtained in the third experiment (Figure 1c), which was conducted by using the inoculum of the previous reactor, sampled during the exponential phase. Indeed, in this experiment, no lag phase was observed, and the maximum growth rate was reached already between day 1 and day 2. Moreover, the growth rate value was 0.0158 g_TSS_/L h. On day 3, the biomass concentration reached the 80% of the maximum achieved concentration, with a 98% increase compared to test 1 and 92% increase compared to test 2. Such observations confirm that by inoculating reactors with microorganisms from the exponential growth phase, the process duration can be considerably reduced.

This evidence is in agreement with a previous study reporting the effectiveness of using a pure culture of *Rhodobacter capsulatus* inoculum during its exponential growth phase instead of prolonged cultivation conditions [10].

**Figure 2:**
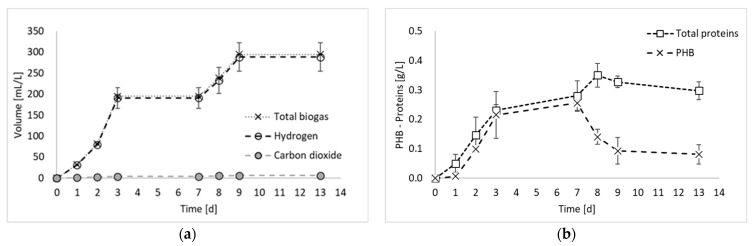

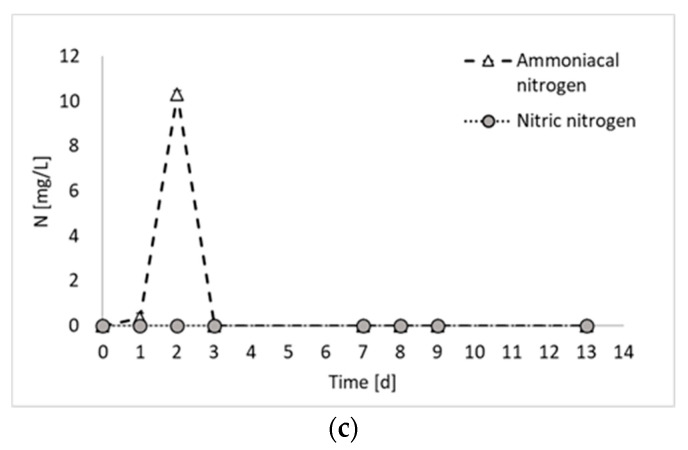
Total biogas, hydrogen, and carbon dioxide production (**a**); microbial product accumulation in terms of total proteins and PHB (**b**); and ammoniacal and nitric nitrogen concentration (**c**) during experiment 3.

Figure 2 reports the results of all products generated during the final hydrogen production test, in terms of total biogas, hydrogen, and PHB, as well as the results of the nitrogen compounds produced during the experiment (i.e., ammoniacal nitrogen, nitric nitrogen, and proteins).

Figure 2a shows that the biogas production started immediately at the beginning of the fermentation period, with an increased rate between days 2 and 3. From day 0 to day 3, a contextual increase in microbial products (i.e., PHB and proteins) was detected, confirming that, as observed previously [8], PPB are involved in both anabolic and catabolic reactions during the first days of fermentation. From day 3 to day 7, no hydrogen was detected, whilst a slight increase in PHB and proteins was observed. This period corresponded to a decrease in the biomass production rate, which has been already observed in previous PF experiments [8] and attributed to a biomass adaptation to a substrate shift in mixed substrate environments. In the present study, only ethanol has been used as carbon source. However, it has to be taken into account that PPB produces and successively consumes organic acids. Therefore, even if a single substrate medium is used, they are always subjected to the presence of mixed carbon sources [9]. Another explanation may be due to the multiple nitrogen sources. In fact, in Figure 2c, it is possible to notice that part of the nitrogen (i.e., 17%), which was initially present in organic form in the medium, was converted into ammoniacal nitrogen during the initial days of fermentation. The ammoniacal nitrogen was subsequently consumed; so, even from the nitrogen substrate point of view, the bacteria were subjected to the presence of multiple forms of nitrogen and a change in their metabolism. In contrast, this phenomenon regarding the cessation of hydrogen production for a period of the fermentation test has not been observed in previous studies using ethanol and glutamate as carbon and nitrogen sources [11]. The discrepancy, however, can certainly be attributed to the nature of mixed culture processes, in which different species coexist and dominant microorganisms may not be the same in different studies.

During the final phase of the process (after day 7), hydrogen was produced again. This result is in accordance with previous studies reporting hydrogen production even at the beginning of the stationary phase [12]. After day 9, hydrogen was not produced anymore. In this final process phase, a decrease in the PHB accumulation was observed, as it was consumed for biomass maintenance. Indeed, in accordance with the feast–famine theory [13], PPB use PHB as a carbon reserve for their survival when nutrients become scarce. Consistently with this observation, the decrease in PHB corresponded to an increase in protein production, which reached the maximum concentration during this final phase of the process.

As already mentioned, ammoniacal nitrogen was produced in the initial days of fermentation. Conversely, the presence of nitric nitrogen has never been detected. The production of ammoniacal nitrogen from glutamate stems from the oxidative deamination process of glutamate, catalyzed by glutamate dehydrogenase, wherein the amino group is removed from glutamic acid and converted into ammonia [14]. This phenomenon aligns with findings from previous studies on photofermentation. For example, Kim et al. (2012) noticed a decline in nitrogenase activity coinciding with an increase in ammonium ion concentration, as ammonia ions resulting from glutamate deamination may inhibit nitrogenase activity [15]. A study by Hillmer and Gest (1977) similarly reported such outcomes, where excess glutamate underwent partial deamination and ammonium ions were detected in the medium using the photosynthetic bacterium *Rhodopseudomonas capsulata* [16]. In contrast to the mentioned studies, which did not observe the depletion of ammoniacal nitrogen after its production, the results of the present study show the complete degradation of ammonium after day 3. Most likely, the presence of mixed cultures facilitated the assimilation of diverse nutrient sources.

**Figure 3:**
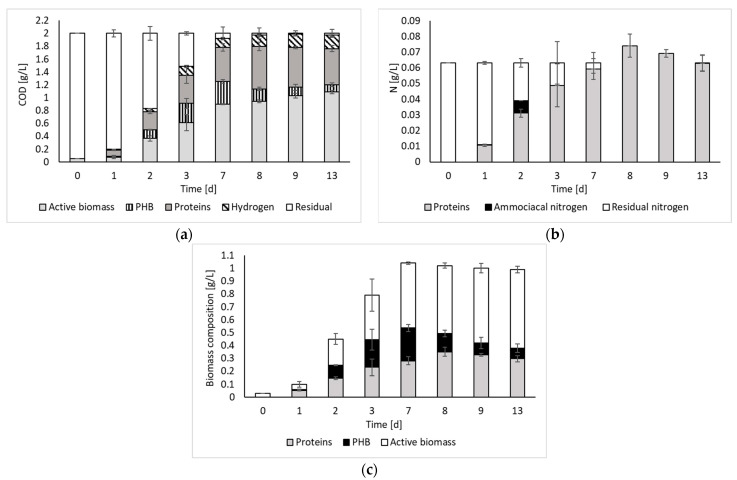
COD (**a**) and nitrogen (**b**) balances, and biomass composition (**c**) over time.

Figure 3 reports the results of the COD and nitrogen balances, as well as the biomass composition over time.

The COD balance indicates that, after day 7, all the initial substrate was converted to the detected products. Such results agree with previous works reporting a stable PF effluent at the end of the process [8]. Indeed, if no inhibition occurs, all the organics contained in the medium (i.e., organic substrates and intermediate organic acids) can be converted to fermentation products.

The COD balance also indicates that, despite the process being capable of producing hydrogen, most of the input substrate was converted to biomass and its associated products (i.e., PHB and proteins). Therefore, the contextual recovery of both energy and biomass products, which is paramount in the framework of a biorefinery concept, can be pursued. Considering the biomass composition, it is possible to observe that both the protein and the PHB contents varied during the fermentation period, although they usually represented approximately 50% of the biomass composition. The remaining part, which still represents a significant fraction, has been identified as active biomass (other than proteins and PHB). According to the literature, this part of the biomass, besides containing microbial cell constituents, may also contain bacteriochlorophylls and carotenoids [4]. These represent other important products worth investigating as a perspective of this study. Indeed, according to the literature, these compounds have significant relevance in various industrial applications (e.g., food, pharmaceutical, and cosmetic sectors). For instance, carotenoids are commonly used as coloring agents in food and as additives in cosmetics, whereas bacteriochlorophylls show promise as chemical compounds for photodynamic therapy [17].

Regarding the nitrogen balance, it was possible to observe that, during the fermentation process, nitrogen was progressively transformed into proteins. Only on day 2, the presence of ammoniacal nitrogen was noticed. However, it was immediately consumed. Beyond day 7, residual nitrogen was no longer present. However, the amount of nitrogen calculated from protein measurements appears to be overestimated, as the balance does not close with an excess of nitrogen of 17% and 9% on days 8 and 9, respectively. This could be due to the fact that the estimate was made indirectly, using albumin as reference.

Table 1 reports the data regarding the production of hydrogen, energy, PHB, and proteins relative to the days of their maximum production. The day of maximum biomass growth rate was also considered. Additionally, the average estimated economical gains potentially obtainable from the recovery of the products have been reported.

The cumulative production of hydrogen was optimized towards the end of the fermentation process (day 9). This day corresponded to the lowest value of PHB production and a high production of proteins, although it was not the highest value observed. Considering the maximum PHB production, it was reached on day 7, which, however, corresponded to low values of hydrogen and protein production. The maximum protein production was instead achieved on day 8, which corresponded to intermediate production levels of both PHB and hydrogen. 

Considering the potentially obtainable revenue, the latter was maximized on day 8 at USD 3423 per cubic meter of working volume, mainly due to the high market value of proteins. On this day, proteins and PHB represented 34% and 14% of the dry weight, respectively, totaling 48% of the biomass weight. Comparing these results with the literature reveals that PHB values are lower than in previous studies conducted with pure cultures and in line with studies conducted with mixed cultures [9,18]. This result is due to the nature of mixed cultures, which contain a multitude of species, of which only some may be specialized in PHB accumulation. Regarding protein accumulation, the limited presence of studies in the literature reporting protein production in the mixed culture photo fermentation process prevents a comparison. The maximum protein percentage of 34% is lower compared to studies conducted under photoorganoheterotrophic conditions. Indeed, previous studies reported that the protein content of PPB can vary between 45% and 75% [19]. This result was predictable, since, in this study, the process was conducted under photo fermentative conditions instead of photoorganoheterotrophy. Nevertheless, the observed percentages of proteins and PHB suggest that the biomass obtained, even under these conditions, possesses a good quality to be utilized for the recovery of these valuable products.

Based on the findings of the biomass growth study (Figure 1), it became evident that employing an exponentially growing inoculum is advantageous for the PF process. Moreover, since all outputs were generated simultaneously during the initial fermentation phase (Figure 2), the analysis also included the day corresponding to the maximum biomass production rate, which was observed on day 3. From the table, it is possible to observe that, in terms of rates, all outputs are maximized during the exponential growth phase of biomass (on day 3). Thus, this observation allows us to state that the best strategy to contextually produce hydrogen and valorize the PPB biomass would be to interrupt the batch process on day 3 and then repeat it using the exponentially growing inoculum. An advantageous solution may be to conduct the process in semi-continuous or continuous mode, using an HRT value of about 3 days (when the exponential phase is reached). Furthermore, even considering the composition of the biomass (Figure 3), it was found to be better on day 3, being composed of a maximized total intracellular compound percentage of 56%, of which 27% is PHB and 29% is proteins. In these conditions, daily revenue is maximized and amounts to USD 0.6/m^3^ d. Currently, semi-continuous mode has been identified in previous studies conducted on pure cultures for hydrogen production as the best feeding mode [18]. However, semi-continuous studies in which proteins and PHB are also taken into account have not yet been conducted, neither with pure cultures nor with mixed cultures, and represent the perspectives of this work.

## 3. Materials and Methods

### 3.1. Substrate Composition and Inoculum Enrichment

The substrate was a synthetic medium, containing ethanol (2 gCOD/L) as a carbon source, organic nitrogen in the form of sodium glutamate (442 mg/L), and yeast extract (Sigma-Aldrich, Buchs, Switzerland) (300 mg/L) as a nitrogen source, as reported in previous photo fermentation experiments [11]. Pure chemicals and double-distilled water were utilized. The initial pH of the culture medium was set to 6. Subsequently, the pH was left uncontrolled. The initial inoculum was a mixed culture, originating from a lab-scale tubular photo bioreactor treating cheese whey for photo fermentative semi-continuous hydrogen production. Such a reactor was started up under non-aseptic conditions, and the culture was characterized by the presence of both dark- and photo-fermentative bacteria, with *Rhodopseudomonas* sp. Strain BR0Y6 as the dominant strain [20]. To study the inoculum enrichment as well as the contextual production of hydrogen, PHB, and microbial proteins, the following sequential batch experiments were conducted:

1. Biomass growth test, which was aimed at determining the time required for microorganisms to reach the exponential growth phase under photo fermentative conditions;
2. Inoculum production test, which was performed using a sample from test 1 as an inoculum. This test was aimed at extracting an inoculum of microorganisms in the exponential growth phase (i.e., during the maximum biomass production rate in terms of g_TSS_/L h);
3. Hydrogen production test, in which the inoculum obtained in the previous steps was employed for a final photo fermentation test, aimed at hydrogen production.

During test 3, the PHB and microbial protein contextual production were evaluated, as well. Each test was conducted inoculating the reactors with 2% volume/volume inoculum. All tests were conducted in triplicate, under the operating conditions reported in the following paragraphs. Tests were stopped when hydrogen was not produced anymore. In detail, experiments 1 and 3 had a total duration of 14 days, whilst experiment 2 had a total duration of 10 days. All results were calculated as mean ± standard deviation and the statistical analysis was performed using Microsoft Excel 2021.

### 3.2. Experimental Setup

The experimental setup employed transparent borosilicate glass bottles (Simax, Sazava, Czech Republic) with a 500 mL capacity and a 400 mL working volume. The screw tops made of PVC were appropriately adapted with tubing to facilitate gas and liquid extraction procedures. A batch feeding strategy was implemented, and both liquid and gas samples were analyzed daily to measure biogas volume, hydrogen and carbon dioxide percentage, PHB, proteins, ammoniacal nitrogen, and nitric nitrogen. The photo fermentative operating conditions included room temperature (25 ± 2 °C), uncontrolled pH, and non-sterile environments. To establish anaerobic conditions and eliminate nitrogen gas from the headspace, the reactors were subjected to 20 min argon flushing before usage. The reactors were positioned on magnetic stirrers with the stirring rate set at 250 rpm. Continuous illumination at 4000 lux was maintained through flexible LED strips.

### 3.3. Analytical Procedures

Biogas production was measured using the water displacement method, as outlined in the study by Ghimire et al. (2015) [21]. Subsequently, a Varian Star 3400 gas chromatograph equipped with a ShinCarbon ST 80/100 (Varian Inc., Palo Alto, CA, USA) column and a thermal conductivity detector, with argon as the carrier gas, was employed for gas chromatographic analysis to determine the gas composition. Biomass growth was measured through the Optical Density (OD) method at a 660 nm wavelength, linking total suspended solids (TSSs) concentration with a standard calibration curve. The curve was obtained using a Photolab Spektral spectrophotometer (6600 UV vis) from WTW (Weilheim, Germany).

PHB analysis followed the method described by Oehmen et al. [22]. In detail, in 16 mm glass cuvettes, 1 mL of effluent from the reactor was lyophilized at −40 °C and 0.1 mbar for at least 10 h. Subsequently, 2 mL of chloroform and 2 mL of an acidified methanol solution (10% volume sulfuric acid) with benzoic acid as an internal standard were added. The cuvettes were agitated and heated to 100 °C for 2 h. Afterward, they were cooled to room temperature, and 1 mL of ultrapure water was added. After mixing, a settling time of 1 h was allowed to achieve phase separation. The chloroform (bottom) phase was then transferred to another cuvette, dried with granular sodium sulfate, and separated from the solid phase to be analyzed [22]. PHB concentration was determined using gas chromatography–mass spectrometry (GC-MS) with a ZB Semi Volatiles Zebron column (Phenomenex, Bologna, Italy) and helium as the carrier gas. 

Protein content was measured using the Lowry method [23].

Ammoniacal nitrogen levels were assessed utilizing the colorimetric method, following the standard methods procedure (APHA, 2017). Measurements of nitrates and nitrites were conducted through ionic chromatography, employing a Metrohm 761 Compact Ion Chromatograph (Formello, Italy) equipped with a Dionex (Sunnyvale, CA, USA) IonPac AS12A 4 × 200 mm column. The light intensity was verified using a Lutron-LX-107 (Cuneo, Italy) light meter.

### 3.4. COD and Nitrogen Balance: Economic Evaluation

The COD balance over time was conducted considering the substrate COD of 2 g/L as an input and the COD fractions from hydrogen, PHB, proteins, and active biomass as an output. The difference between the input COD and the sum of all outputs was indicated as residual COD and it included the unconverted substrate and intermediate catabolic products. The products’ COD aliquots (i.e., ${COD}_{product}$ [g/L]) were calculated as follows:

(1) $${COD}_{product}=C_{product}⨯Theoretical\;{COD}_{product}$$

where $C_{product}$ is the concentration of the product in g/L, and the $Theoretical\;{COD}_{product}$ (gCOD/gproduct) is calculated considering the reaction of complete oxidation of the product. To calculate the PHB theoretical COD, the 3HB monomer formula was considered (i.e., C_4_H_8_O_3_). The albumine chemical formula (C_53_H_6.98_N_16_S_1.84_) was used for the calculation of the proteins’ COD. Concerning the active biomass, its concentration was assumed to be the concentration of TSS excluding PHB and proteins. The theoretical COD was calculated considering the *Rhodopseudomonas palustris* elemental molar composition (CH_1.92_N_0.17_O_0.41_) [24].

The nitrogen balance was performed considering the nitrogen content in sodium glutamate and the nitrogen content in yeast extract as inputs. For yeast extract, a nitrogen content of 10.5% was assumed, as indicated on the technical data sheet.

The nitrogen output was determined by summing the nitric, ammoniacal, and theoretical nitrogen from protein, taking into account the chemical formula of albumin for proteins.

For the economical evaluation of products, the market prices of microbial proteins and PHB (i.e., USD 7.76/kg and USD 4/kg, respectively) were considered [25,26].

Concerning the hydrogen market value, the 2023 Italian market price of energy of USD 0.17/kWh was taken into account. Hydrogen energy was calculated from the cumulative hydrogen volume, using the lower heat value of 12.7 J/mL hydrogen [27].

## 4. Conclusions

PPB represent one of the most interesting microorganism groups due to their versatility in metabolism, allowing them to convert organic substrates into a variety of valuable products. The results obtained in this study showed that, under photo fermentative conditions aimed at hydrogen production, it is possible to contextually obtain a valuable biomass with a high content of PHB and proteins. The productivity and preliminary economic analysis showed that a striking strategy to concomitantly optimize these three products is to conduct the process during the exponential growth phase of the biomass. Therefore, the recommendations of this study include to conduct the process under repeated-batch or semi-continuous/continuous mode, using the day of the maximum biomass production rate as the hydraulic retention time. Further studies will also explore the overall product portfolio, likely including additional value-added compounds like bacteriochlorophylls and carotenoids in order to better discuss process feasibility at a larger scale.

## Figures and Tables

**Table 1:** Results analysis in terms of production and economical optimization.

	Optimized Output			
	Hydrogen	PHB	Proteins	Biomass Rate
Day	9	7	8	3
Hydrogen volume (L/m^3^)	288 ± 34	190 ± 24	233 ± 30	191 ± 25
Energy H^2^ (kWh/m^3^)	1.03 ± 0.12	0.68 ± 0.30	0.83 ± 0.40	0.68 ± 0.30
Hydrogen rate (L/m^3^ d)	32.07 ± 3.78	27.28 ± 3.49	29.15 ± 3.47	63.63 ± 8.30
Energy rate (kWh/m^3^ d)	0.11 ± 0.12	0.10 ± 0.08	0.10 ± 0.10	0.23 ± 0.10
PHB (kg/m^3^)	0.14 ± 0.04	0.26 ± 0.02	0.14 ± 0.02	0.13 ± 0.06
PHB (%)	0.09 ± 0.04	0.25 ± 0.02	0.14 ± 0.02	0.21 ± 0.02
PHB rate (kg/m^3^ d)	0.015 ± 0.004	0.036 ± 0.004	0.018 ± 0.004	0.045 ± 0.006
Proteins (kg/m^3^)	0.338 ± 0.010	0.280 ± 0.030	0.350 ± 0.034	0.147 ± 0.020
Proteins (%)	0.327 ± 0.012	0.270 ± 0.030	0.343 ± 0.034	0.230 ± 0.020
Proteins rate (kg/m^3^ d)	0.038 ± 0.002	0.040 ± 0.004	0.044 ± 0.004	0.049 ± 0.006
Revenue_Energy ($/m^3^; $/m^3^ d)	0.174 ± 0.020; 0.019 ± 0.002	0.115 ± 0.014; 0.016 ± 0.002	0.141 ± 0.018; 0.018 ± 0.002	0.115 ± 0.014; 0.038 ± 0.004
Revenue_PHB ($/m^3^; $/m^3^ d)	0.550 ± 0.178; 0.061 ± 0.020	1.022 ± 0.111; 0.146 ± 0.016	0.563 ± 0.103; 0.070 ± 0.012	0.540 ± 0.103; 0.180 ± 0.003
Revenue_Proteins ($/m^3^; $/m^3^ d)	2.63 ± 0.07; 0.292 ± 0.010	2.174 ± 0.18; 0.311 ± 0.034	2.719 ± 0.20; 0.340 ± 0.032	1.141 ± 0.20; 0.380 ± 0.060
Total revenue ($/m^3^; $/m^3^ d)	3.355 ± 0.266; 0.373 ± 0.029	3.31 ± 0.307; 0.473 ± 0.043	3.423 ± 0.322; 0.428 ± 0.040	1.796 ± 0.310; 0.599 ± 0.103

## Data Availability

Data will be available on request.
